# Ochronotic Deposition in Alkaptonuria: Semiquinone-Mediated Oxidative Coupling and Metabolic Drivers of Homogentisic Acid Accumulation

**DOI:** 10.3390/ijms26199674

**Published:** 2025-10-03

**Authors:** Daniela Grasso, Valentina Balloni, Maria Camilla Baratto, Adele Mucci, Annalisa Santucci, Andrea Bernini

**Affiliations:** 1 Department of Biotecnology, Chemistry and Pharmacy, University of Siena, 53100 Siena, Italy; 2 Department of Chemical and Geological Sciences, University of Modena and Reggio Emilia, 41125 Modena, Italy

**Keywords:** alkaptonuria, ochronotic pigment, homogentisic acid, semiquinones, oxidative coupling, nuclear magnetic resonance, electron paramagnetic resonance

## Abstract

Alkaptonuria (AKU) is a rare metabolic disorder caused by homogentisate 1,2-dioxygenase (HGD) deficiency, leading to homogentisic acid (HGA) accumulation and ochronotic pigment deposition, which drug therapy cannot reverse. The process of pigment formation and deposition is still unclear. This study offers molecular insights into the polymeric structure, with the goal of developing future adjuvant strategies that can inhibit or reverse pigment formation, thereby complementing drug therapy in AKU. HGA polymerisation was examined under physiological, acidic, and alkaline conditions using liquid and solid phase nuclear magnetic resonance (NMR), electron paramagnetic resonance (EPR), and polyacrylamide gel electrophoresis. At physiological pH, HGA polymerised slowly, while alkaline catalysis accelerated pigment formation while retaining the HGA aromatic scaffold. During the process, EPR detected a semiquinone radical intermediate, consistent with an oxidative coupling mechanism. Reactivity profiling showed the diphenol ring was essential for polymerisation, while –CH_2_COOH modifications did not impair reactivity. Pigments displayed a polydisperse molecular weight range (11–50 kDa) and a strong negative charge. Solid-state NMR has revealed the presence of phenolic ether and biphenyl linkages. Collectively, these identified structural motifs can serve as a foundation for future molecular targeting related to pigment formation.

## 1. Introduction

**Figure 1:**
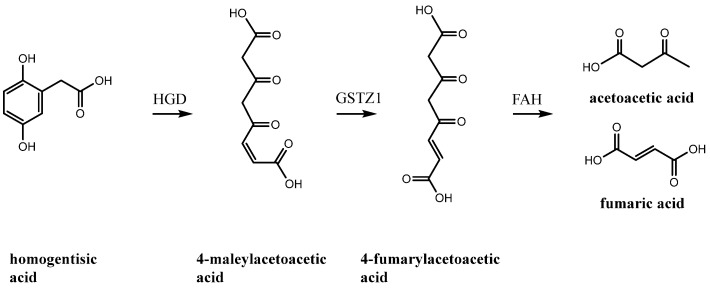
Degradation pathway of homogentisic acid. The step affected in alkaptonuria is the ring opening of homogentisic acid (**1**) by homogentisate-1,2-dioxygenase (HGD), which, in pathological variants, prevents the enzyme from processing the substrate to 4-maleylacetoacetic acid. The substrate then accumulates and transforms into ochronotic pigment. HGD: homogentisate 1,2-dioxygenase; GSTZ1: glutathione S-transferase zeta 1; FAH: fumarylacetoacetate hydrolase.

Alkaptonuria, a rare autosomal recessive disorder (OMIM: 203500), holds a significant place in the history of genetics [1]. It was the first human disorder recognised to conform to the principles of Mendelian autosomal recessive inheritance [2,3], a milestone established by Sir Archibald Garrod over 100 years ago. In his Croonian Lecture at the Royal College of Physicians in 1908, Garrod introduced the concept of “inborn errors of metabolism” to describe AKU and three other inherited disorders, albinism, cystinuria, and pentosuria. This pioneering work laid the foundation for our understanding of genetic diseases, with Garrod’s belief that diseases resulted from missing or faulty steps in the body’s chemical pathways, and his introduction of the concept of ‘chemical individuality’, predating the discovery of ‘genes’ by more than 100 years. According to a comprehensive report from 2020 [4], there are 1233 known patients globally, with certain regions, such as Slovakia and the Dominican Republic, exhibiting significantly higher rates than the average. The term alkaptonuria is derived from the Arabic word “alkali”, and it was introduced by Boedeker in 1859 when he noted the distinctive dark colour and reducing property of urine in an AKU patient. In 1866, Virchow [4] first described and named ochronosis after witnessing a dark pigment under a microscope, which he described as ochre. The compound responsible for this dark colouration was identified by Baumann and Wolkow in 1891, and it was named “alcapton”, a byproduct of homogentisic acid oxidation. Homogentisic acid (HGA), also referred to as 2,5-dihydroxyphenylacetic acid, is an intermediate product in the degradation of phenylalanine and tyrosine. Under normal physiological conditions, these amino acids are metabolised to fumaric and acetoacetic acids through a series of enzymatic reactions (Figure 1), wherein the aromatic ring is cleaved and oxidised by homogentisate-1,2-dioxygenase (HGD) [5].

AKU arises from the loss of activity of the enzyme HGD. The HGD mutation database has recorded 253 unique variants of the HGD gene (http://hgddatabase.cvtisr.sk/, accessed on 10 January 2023) [6] derived from DNA sequencing, with missense mutations being the most prevalent, representing 139 unique cases (55%). This deficit results in accumulations of HGA in the blood. To reduce the high concentration, the kidneys eliminate HGA, resulting in gram quantities in the urine, which turn dark upon contact with air.

The process that transforms HGA into ochronotic pigment through the creation of a large, inflexible molecule at the nanometre scale remains unclear, although it is thought to resemble melanin pigment formation: melanin synthesis begins with the amino acid tyrosine, which is converted by the enzyme tyrosinase into DOPA and then into dopaquinone; from dopaquinone, the pathway leads to the production of eumelanin (brown/black pigment), mostly made of indole-5,6-quinone and 5,6-dihydroxyindole-2-carboxylic acid units, polymerised into a dark, insoluble material [7].

From the third to the fourth decade of life, severe consequences of the ochronotic process are observed, such as valvular heart disease, spondyloarthropathy, or rupture of ligaments, muscles, or tendons, highlighting the progressive and debilitating nature of the disease [8,9,10,11].

Alkaptonuria can be treated with the recently approved drug Orfadin. The active molecule is nitisinone, also known by its chemical name NTBC (2-(2-nitro-4-trifluoromethylbenzoyl)-1,3-cyclohexanedione), a molecule discovered initially in the late 1980s during a program aimed at developing new herbicides [12,13]. The potential of nitisinone for treating alkaptonuria was later explored. Studies have shown that by inhibiting HPPD, nitisinone significantly reduces the production of homogentisic acid [14].

Subsequently, based on clinical trial results of the SONIA 2 study [15], which confirmed nitisinone’s efficacy in reducing HGA levels and slowing disease progression, the EMA approved Orfadin for the treatment of adult patients with alkaptonuria in September 2020. However, its use is not without adverse side effects. A significant concern is the resulting increase in plasma tyrosine levels due to the upstream blockade of tyrosine degradation, which can lead to keratopathy, cognitive effects, and potential dermatological issues. Other side effects may include thrombocytopenia, leukopenia, and abnormalities in liver function. Due to these risks, patients undergoing treatment with nitisinone require regular monitoring of tyrosine levels, liver function tests, and comprehensive ophthalmological evaluations. It is also worth noting that nitisinone does not reverse existing damage, such as joint or heart valve damage caused by ochronosis.

Regardless of the introduction of such a therapeutic option for AKU, investigating the ochronosis process is an urgent necessity for the research of adjuvant therapies targeting pigment formation and disruption. To achieve this, a better understanding of the underlying molecular process and the molecular structure of the pigment is required.

Our previous study [16], comparative of AKU urine and HGA solution developing dark pigment, demonstrated a radicalic mechanism underlies the phenomenon. A soluble, unsaturated polymer formed by the catalysis of hydroxyl ions. The size and heterogeneous structure of the polymer prevented further molecular elucidation by NMR.

The present work introduces new pigment separation methods and spectroscopic approaches, such as solid-state NMR, as well as experiments involving molecules with altered functional group properties. This provides further insights into the structural determinants, size, and morphology of the pigment.

## 2. Results and Discussion

### 2.1. HGA Discolouration Under Physiological Conditions

**Figure 2:**
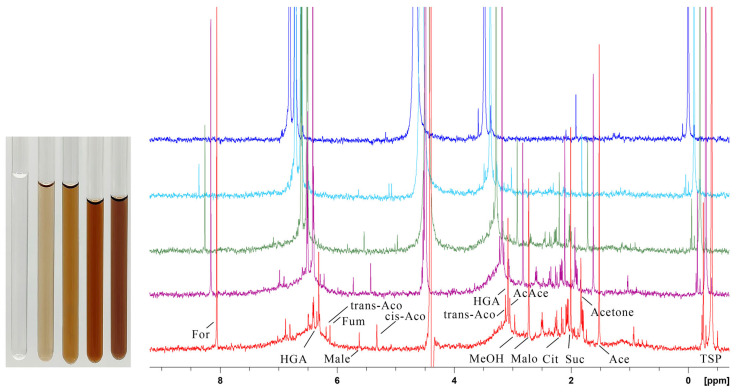
NMR monitoring of HGA reaction under physiological pH and temperature. In the right panel, from top to bottom, stacked spectra at start (blue), after 1 week (pale blue), after 4 weeks (green), after 8 weeks (purple), and after 10 weeks (red) are shown; in the left panel, for each spectrum, a picture of the NMR sample tube is reported for the same intervals, time growing from left to right. Darkening to ochre colour is apparent. The peak area for HGA decreases to 1% after 10 weeks. Opposite to this, sharp peaks arise with time, identified as products of HGA catabolism, acetoacetate (AcAce), fumarate (Fum) and its isomer, maleate (Male); large fragments from ring opening as the tri-carboxylic acids citrate (Cit), cis-aconitate (cis-Aco), and trans-aconitate (trans-Aco); small residual fragments from ring opening: acetone, acetate (Ace), formate (For), malonate (Malo), methanol (MeOH), and succinate (Suc). The chemical shift ppm scale refers to the top spectrum.

Pigment development in biofluids has been demonstrated to be reproducible with HGA solutions of similar concentration [17]. We then investigated pigment formation under physiological conditions in vitro. A 5.0 mM solution of HGA, buffered at physiological pH 7.4, was incubated at 35 °C, and NMR spectra were recorded at intervals (Figure 2).

After 10 weeks, the HGA signal has dropped to 1%, with the peaks of molecules originating from ring breakage contributing an additional 8% (normalised to the number of HGA protons), resulting in a net loss of 91% of the signal area. The only other spectral change is the rise of broad, weak peaks at the base of the HGA signals (see Figure 2 HGA marks). Such an observation would confirm that the HGA scaffold is preserved during polymerisation, although other phenomena are occurring; the origin of the peak broadening will be investigated in the following sections.

### 2.2. HGA Discolouration Under Non-Neutral Conditions

**Figure 3:**
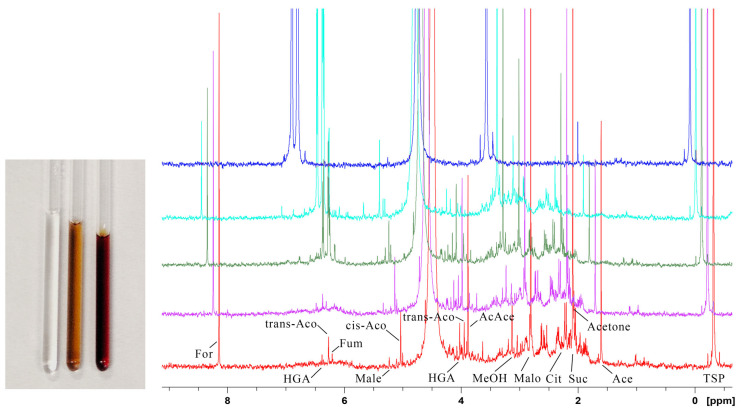
NMR monitoring of HGA reaction under alkaline pH at room temperature. In the right panel, from top to bottom, stacked spectra at start (blue) and after 8 h (pale blue), 24 h (green), 1 week (purple), and 2 weeks (red) are reported; in the left panel, a picture of the NMR sample tube is reported at the start, after 24 h and after 2 weeks, time growing from left to right. Apart from the increased rate, the reaction proceeds with the same byproducts as in Figure 2, although peaks are shifted due to the increased pH. The chemical shift ppm scale refers to the top spectrum.

The discolouration of the HGA solution has been examined under non-neutral conditions. An acidic solution using acetic buffer (pH 4.5) remains stable for one week at 35 °C, with no darkening or changes detected in NMR spectra. On the other side of the pH scale, the impact of alkali on HGA is well established [18]. When NaOH or NH_4_OH is added, the HGA solution instantly turns brown at room temperature, and the consumption of HGA occurs at a significantly accelerated rate, as depicted in Figure 3: the HGA concentration reduces to 6% within 24 h and is undetectable after 2 weeks. Similar results are obtained with NH_4_OH. NMR showed that the reaction under alkaline conditions proceeds with the same byproducts and peak broadening as observed in the HGA reaction at physiological conditions of pH 7.4 and T = 35 °C. Alkalinisation is then a feasible way to obtain the large quantities of pigment required for subsequent analyses while ensuring the complete reaction and avoiding permanence of radical species (see Section 2.3), which hampers NMR investigation.

### 2.3. HGA Polymerisation Mechanism

**Figure 4:**
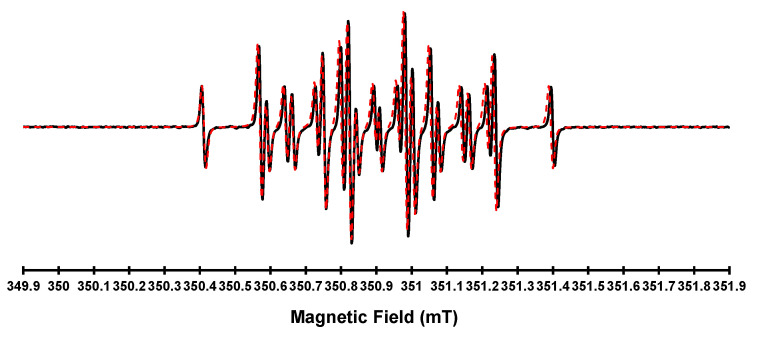
Room temperature (298 K) X-band (9 GHz) EPR spectrum of HGA (1) reacting under alkaline conditions (black line) paired to its simulation (red line). The spectrum was recorded at a microwave frequency of 9.868 GHz, with a microwave power of 2 mW and a modulation amplitude of 0.01 mT. The EPR spectrum was recorded immediately after the addition of an excess of NH4OH to HGA, and it was simulated using the Easyspin program, version 6.0.0, with the garlic function. The radical species was simulated with the magnetic parameter g = 2.0048 ± 0.0001 and the interaction of the unpaired electron with 3H, not magnetically equivalent, with coupling constants of 2.5 G, 2.3 G, and 1.8 G, and 2H, magnetically equivalent, with a coupling constant of 1.6 G.

In a previous work [16], we demonstrated how the pigment development proceeds through radical intermediates by EPR monitoring of the HGA reaction. The exceedingly slow HGA darkening under the physiological condition hampers the detection of radicals by EPR. On the contrary, alkaline catalysis by NaOH generated enough radical species for EPR detection. Still, their rapid evolution limited the structural understanding of the initial paramagnetic intermediates, thereby restricting the use of EPR to monitoring radical extinction and reaction completeness. In this study, we found a trade-off between the two conditions by adding the less harsh NH_4_OH to the HGA solution. It resulted in clear EPR spectra of a single dominant paramagnetic species at the very start of the darkening reaction. The subsequent simulation offers precise parameters to gain structural insight into the original radical (Figure 4).

**Figure 5:**
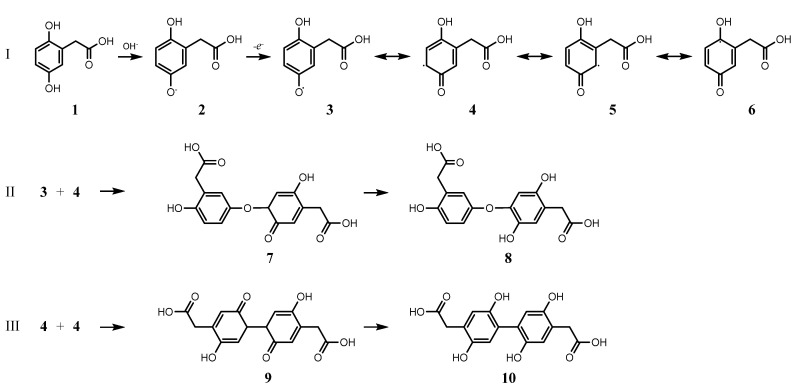
Oxidative coupling pathways for HGA: dissociation of hydroxyl and successive semiquinone formation with delocalisation (I); coupling and rearrangement in diphenolic form, suitable for further reaction (II, III).

The g value and couplings indicate the formation of a semiquinone as a radical intermediate [19,20], suggesting an oxidative coupling-driven polymerisation illustrated in Figure 5: increasing pH promotes the dissociation of hydroxyl in HGA (**2**), which then converts to a semiquinone (**3**), as indicated by EPR. The unpaired electron can further delocalize as shown by the resonance structures **4**, **5**, or **6** (**I**).

We can then identify two primary coupling pathways: **3** may couple with **4**, supported by reduced hindrance at the unpaired electron site (**II**); alternatively, a pair of **4** might join together (**III**). In both scenarios, proton rearrangement produces species capable of further reaction, as two phenolic hydroxyls per ring remain accessible (**8** and **10**), a requirement assessed in Section 2.4. Other potential responses in intermediate reactions include the oxidative aromatic ring cleavage following the addition of dioxygen to radicals **4**, **5**, and **6**, leading to the byproducts observed in the NMR spectrum of the pigment. Dioxygen addition to **6** has indeed been observed in HGD-catalysed HGA oxidation [21]. Moreover, such a reaction could also occur in the condensed phenolic structures of types **8** and **10**.

### 2.4. Discolouration of HGA Similars

**Figure 6:**
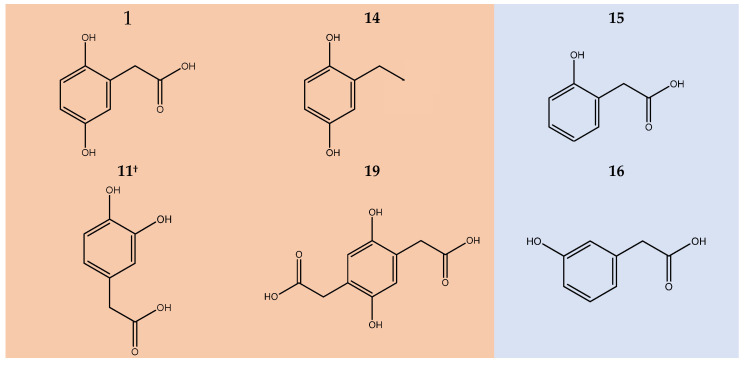

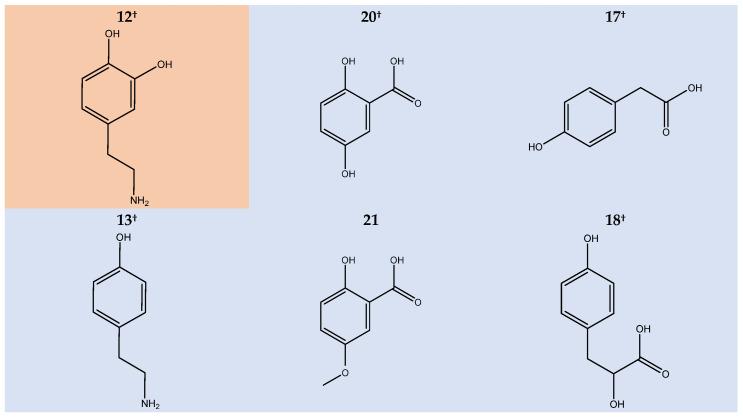
Homogentisic acid and its derivatives: orange background: reactive; pale blue: stable. **^†^** Found in homogentisate metabolism. **1**: homogentisic acid; **11**: 3,4-dihydroxyphenylacetic acid; **12**: dopamine; **13**: tyramine; **14**: 2-ethylbenzene-1,4-diol; **15**: 2-hydroxyphenylacetic acid; **16**: 3-hydroxyphenylacetic acid: **17**: 4-hydroxyphenylacetic acid; **18**: p-hydroxyphenyllactic acid; **19**: 2,5-dihydroxy-1,4-benzenediacetic acid; **20**: gentisic acid; **21**: 2-hydroxy-5-methoxybenzoic acid.

To confirm the oxidative coupling polymerisation and to further investigate the role of aromatic ring substituents in the reaction pathway, we conducted an extensive NMR and EPR study on HGA analogues carrying modified or swapped substituents (Figure 6).

In particular, the involvement of phenolic hydroxy groups was explored by swapping the 5-hydroxyl group with acetate (11), by removing one hydroxyl substituent (15, 16, 17) and reintroducing it as part of a chain substituent (18), and by hydroxyl methylation (**21**). Additionally, the role of the –CH_2_COOH group has been investigated through transamination (**12**, **13**), shortening (**20**), reduction (**14**), and doubling (**19**). Some compounds also belong to the HGA branch of tyrosine catabolism (11, 12, 13, 17, 18, 20), providing insight into potential cross-reactivity among the pathway metabolites. Compounds that exhibit discolouration, NMR signal decrease, and EPR signal upon alkalinization are highlighted in orange in Figure 6, otherwise in pale blue. Spectra are available as Supplementary Materials (Figures S1–S11).

The permutation of the hydroxyl pair from the reciprocal para position to the ortho does not affect the reaction (**11, 12).** On the other hand, the removal of a hydroxy substituent completely inhibits the reaction (**13**, **15**, **16**, **17**), as it does with methylation (**21**). However, reintroducing a hydroxy group to the side chain does not recover activity (**18**). Such observation infers the essential role of the diphenol ring over the other substituents, supporting the hypothesis of a semiquinone-centred reaction. This is strengthened by the observation that –CH_2_COOH group substitution for ethyl (**14**), ethylamine (**12**), or the addition of a second acetate group (**19**) does not hinder reactivity. Interestingly, the hydrocarbon substituent of **14** produces a similarly soluble pigment as HGA, stable in solution for months.

### 2.5. Investigation of Pigment Particles

#### 2.5.1. Gel Electrophoresis Separation of Pigment Components

To attribute the origin of the broad peak along the HGA signal disappearance shown in Figure 2 to the polymer formation process, morphological characterisation of the pigment particles was attempted following the chemical characterisation. Polyacrylamide gel electrophoresis proved effective in characterising the mass distribution of HGA pigment particles. Such a gel, at 15% polyacrylamide, is suitable for separating molecules in the range of 3 kDa to 100 kDa. A sample of HGA pigment was loaded onto the gel and migrated from the well through the running gel without requiring SDS loading buffer, indicating an intrinsic strong negative charge, likely due to the carboxyl group inherited from HGA. Moreover, the dark colour of the pigment enabled us to observe the bands without the need for any stain. SDS and staining were used to run and reveal the bands of the protein ladder, which was included for molecular weight referencing.

**Figure 7:**
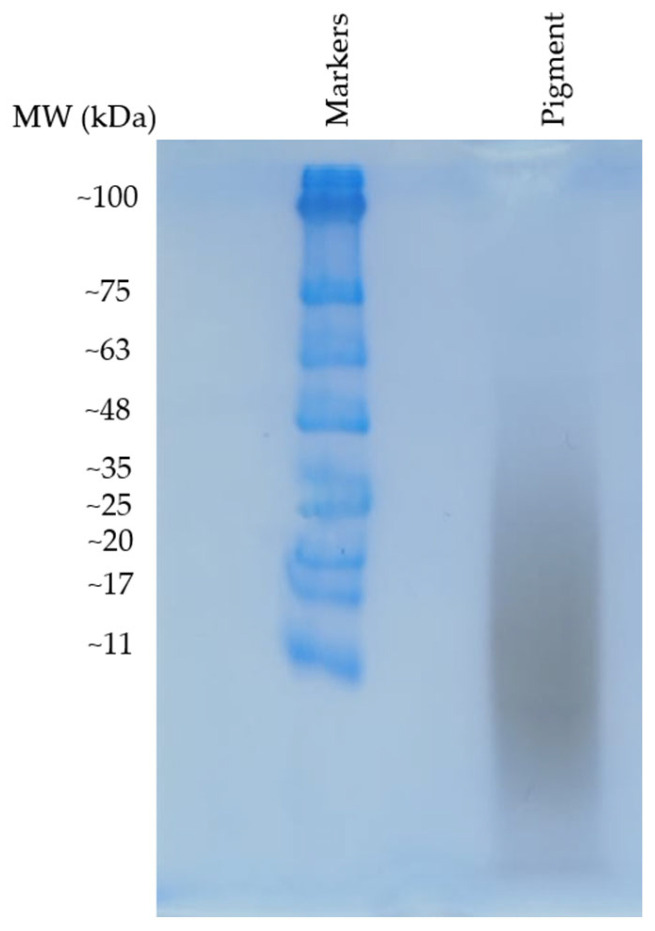
Polyacrylamide gel pattern of the HGA pigment. In the first lane, a protein ladder, spanning from ~11 kDa to ~100 kDa, was used for molecular weight comparison. In the pigment lane, a single broad, dark smear appeared without the use of any stain, thanks to the dark colour of the HGA pigment.

Results (Figure 7) show a diffuse smear was observed, extending approximately to the 48 kDa mark, without resolving into distinct bands. This indicates a broad, polydisperse molecular mass distribution of particles. The polymer’s natural negative charge may have affected its migration, regardless of SDS interaction, possibly resulting in deviations from standard protein migration patterns and making it harder to accurately determine the mass. Nevertheless, the experiment offers a rough idea of the molecular weight distribution, confirming the presence of a polymeric material with molecular weight components centred around 11 kDa and spread up to the 50 kDa range.

#### 2.5.2. Ultrafiltration Separation of Pigment Components

**Figure 8:**
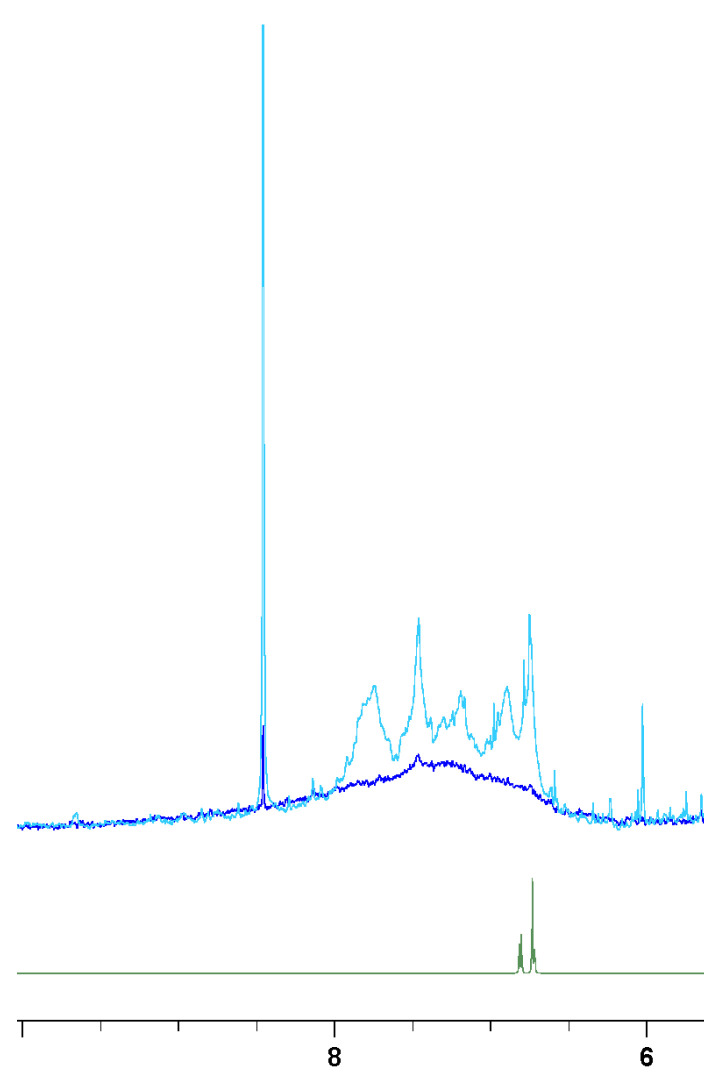
Permeate (pale blue) and retentate (dark blue) of the in vitro reacted dark pigment upon ultrafiltration at a 3 kDa cutoff. The spectrum of unreacted HGA is reported in green as a chemical shift reference. The retentate lost its peak shape, resulting in a broad, extensive peak.

Gel separation does not allow recovery of sufficient material for NMR spectroscopy, so we utilised a proven NMR method to analyse mixtures of biopolymers and metabolites. A concentrated pigment solution, obtained from the complete reaction of a 178 mM HGA with a five-equivalent excess of NH_4_OH, was ultrafiltered using a 3 kDa MWCO membrane; retentate (MW > 3 kDa) and permeate (MW < 3 kDa) were then analysed by solution NMR (Figure 8).

Although very simple, the ultrafiltration process efficiently eliminates low-molecular-weight metabolites and small oligomers (3 kDa corresponds roughly to 18 HGA molecules) while retaining large polymer particles, as indicated by the retentate/permeate ratio for formate (8.44 ppm) of less than 0.7%. Permeate shows a complex of broad but equally dispersed aromatic peaks, possibly reflecting the heterogeneity of low-molecular-weight reaction intermediates hypothesised in Figure 5. Retentate (particles MW > 3 kDa) shows a more dramatic broadening with a single wide, weak signal spanning the full aromatic region. The ultrafiltration experiment was repeated with a 100 kDa MWCO membrane at the same concentration in search of very high-molecular-weight components, but the retentate showed no NMR signals, in agreement with the results of gel electrophoresis, which reports no particles at MW > 100 kDa. Polymeric chains, despite their high molecular weight, give rise to defined peaks in solution NMR, provided that segmental/rotational mobility is allowed (e.g., polyvinyl and, to a minor extent, cellulose [22,23,24]). This does not appear to be the case for HGA polymer, which exhibits exceedingly broad peaks. The combined effects of polymer chain rigidity (see oligomers 26 and 28) and heterogeneity in chain elongation (due to the cross-combination of pathways of types II and III), may lead to the significant signal broadening observed in HGA polymer, even at limited chain elongation; this is supported by the fact that the particle size does not appear to exceed 50 kDa in MW (as indicated by gel separation and ultrafiltration) nor surpass 10 nm in hydrodynamic radius (as seen in earlier dynamic light scattering measurements [6]).

However, the excessive peak broadening of the retentate polymer hinders further structure elucidation by solution NMR.

#### 2.5.3. Solid-State NMR of the Pigment

**Figure 9:**
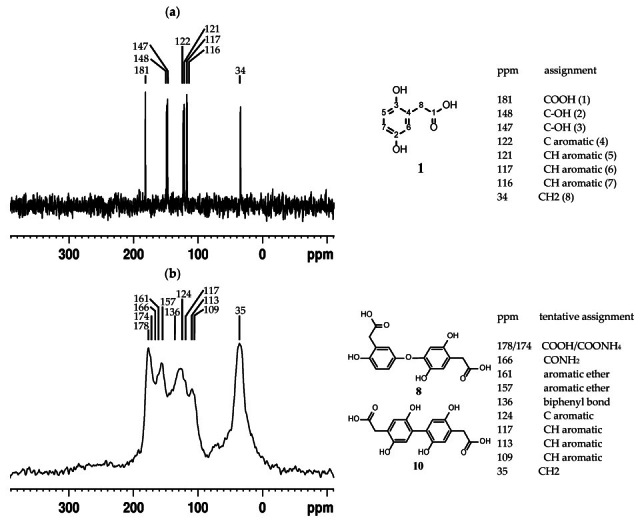
^13^C CP-MAS NMR spectra of (**a**) compound **1** (HGA) with assignment; (**b**) pigment MW > 3 kDa, with tentative assignments of existing and new functional groups.

Excessive peak broadening hinders structure elucidation by solution NMR, as shown in the previous section. We then lyophilised the pigment fraction > 3 kDa obtained from ultrafiltration and analysed the powder by solid-state NMR to investigate its molecular components. By using ultrafiltered pigment retentate, we ensure that no interference arises from unreacted HGA, small-molecule byproducts, or small oligomers, but only from the heavier particles observed in gel chromatography. To identify any significant chemical shift changes in carbon signals that might indicate involvement in polymer formation, the ^13^C resonances of the pigment powder were compared to those of solid HGA (see Figure 9 for spectra and assignments).

A previous study demonstrated that an alkaline environment alone does not alter the 13C spectrum of HGA [17], while peaks for the pigment were confirmed by heteronuclear correlation NMR experiments (see Supplementary Material Figure S12). In the upfield half of the ^13^C axis (0–125 ppm), the group formed by CH_2_ (8), aromatic CHs (5, 6, 7), and the quaternary carbon (4) does not experience a significant signal shift upon polymerisation. On the contrary, the phenolic carbons (2, 3) seem to undergo parallel ~10 ppm deshielding (from 147/148 ppm to 157/161 ppm), typical of phenolic ether formation. Such a transformation would correlate with the ether group shown by the intermediate molecule family of type **26**. Between the two spectral groups, an original peak arises in the pigment spectrum, with a chemical shift (136 ppm) typical of quaternary carbon at biphenyl bonds. Such an observation would correlate with the straight ring–ring bond shown by molecule **28.** At the far end of the NMR spectrum, the COOH peak at 181 ppm splits into peaks at 178, 174, and 166 ppm. This suggests the formation of an ammonium salt and an amide from the carboxy group, resulting from the use of ammonia as a basic catalyst, rather than from its participation in the polymer formation process.

Solid-state NMR is the final step in the spectroscopic investigations that lead to a heterogeneous polymeric structure. However, this method has the limitation that hydroxyl catalysis may affect particle size due to the accelerated reaction rate. Another limitation of the spectroscopic workflow is that to fine-tune the molecular mechanism, other biomolecules, such as proteins and lipids, which may participate in secondary reactions, have been excluded from the environment. These biomolecules will need to be further investigated in simplified tissue-mimicking studies. Nevertheless, the structural mechanism motifs identified here align with the overall macroscopic outcome of ochronosis, while these structural motifs may provide molecular targets for additional therapies.

## 3. Materials and Methods

### 3.1. Solution NMR

Samples of 5 mM in water were prepared for all the compounds under investigation (see Section 2.2). Deionised water was purified using a Milli-Q^®^ System from Millipore (Burlington, MA, USA). For chemical shift and peak area calibration, the 3-(trimethylsilyl)-2,2,3,3-tetradeuteropropionic acid (TSP, Merck KGaA, Darmstadt, Germany) to a final concentration of 0.5 mM was used. The HGA sample under physiological conditions was buffered to pH 7.4 with a 50 mM phosphate buffer, maintained at T = 35 °C in an incubator and monitored at start and at weeks 1, 4, 8, and 10. The HGA sample under acidic conditions was buffered to pH 4.5 with 50 mM acetate buffer, maintained at T = 35 °C in an incubator and monitored at the start and at week 1. Samples of all compounds under alkaline conditions were basified using 12 µL of either NaOH or NH4OH 10 M and monitored by NMR after 8 h, 24 h, 1 week, and 2 weeks.

All experiments were performed on a Bruker Avance™ 600 spectrometer (Bruker Biospin, Rheinstetten, Germany) operating at 14.1 T using a 1D-1H PURGE pulse sequence. Data processing, peak identification and compound quantitation were carried out with the TopSpin 4.0.8 software (Bruker). All the 1D-1H spectra were acquired with a spectral width of 6 MHz and 32 scans, digitised over 32 k points and zero-filled to 64 k. Solvent signal removal was achieved with a pre-saturation power of 55 dB during repetition delay (4 s).

### 3.2. Compounds

The following compounds were tested by NMR for discolouration under alkaline conditions (Merck KGaA): homogentisic acid; 3,4-dihydroxyphenylacetic acid; dopamine; tyramine; 2-ethylbenzene-1,4-diol; 2-hydroxyphenylacetic acid; 3-hydroxyphenylacetic acid; 4-hydroxyphenylacetic acid; p-hydroxyphenyllactic acid; 2,5-dihydroxy-1,4-benzenediacetic acid; gentisic acid; 2-hydroxy-5-methoxybenzoic acid.

### 3.3. EPR Spectroscopy

Continuous wave X-band (9 GHz) EPR spectra of the samples were recorded at room temperature. The reaction was monitored immediately after the addition of alkali to compounds, as in Section 2.1. EPR measurements were performed with a Bruker E580 Elexsys Series using the Bruker ER4122SHQE cavity filling in a 1 mm ID quartz capillary tube, and then it was placed inside a standard suprasil EPR tube (3.5x4 IDxOD). EPR spectrum simulation was performed with the Easyspin software package, version 6.0.0, using the garlic function.

### 3.4. SDS-PAGE Electrophoresis Buffers

The stacking gel was prepared with 4% acrylamide, while the running gel was prepared with 15% acrylamide (see Table 1 for details). A molecular weight standard was included for comparison (MaestroGen, Hsinchu City, Taiwan, AccuRuler RGB prestained ladder). Electrophoresis was carried out at 200 V for 30 min. The dark colour of the pigment allowed us to visualise the bands without using any stain. However, the gel was stained with Coomassie Brilliant Blue stain to visualise the marker bands.

### 3.5. Solid State NMR

NMR spectra were acquired using an AVANCE III HD 600 Bruker spectrometer equipped with a 2.5 mm H/X CPMAS probe operating at 600.13 MHz for ^1^H and at 150.90 MHz for ^13^C. Zirconia rotors of 2.5 mm o.d. were used and spun at 16 kHz magic-angle spinning (MAS) rates. ^1^H MAS NMR spectra were acquired using DEPTH sequence in order to remove baseline distortions. A cross-polarization (CP) sequence was used to acquire ^13^C spectra. The parameters used for ^1^H NMR spectra were 125 kHz spectral width, 3 s relaxation delay, 2.5 μs P90 pulse, 4 k data points, and 32 scans. The parameters used for CP-MAS ^13^C NMR spectrum were 76 kHz spectral width, 2 s relaxation delay, and 2 k data points. The FID from two experiments with 0.5 and 1 ms contact time were added for a total of 51,200 scans. Two C,H hetero-correlated frequency-switched Lee–Goldburg (FSLG) pulse sequence 2D NMR experiments with 0.2 and 1 ms contact time were done to distinguish CH_n_ from quaternary carbons [25].

All chemical shifts were referenced by adjusting the spectrometer field to the value corresponding to 38.48 ppm chemical shift for the deshielded line of the adamantane ^13^C NMR signal. With this setting, the TOPSPIN v 4.08 software (Bruker) generates data sets with the peak positions correctly calibrated according to IUPAC recommendations [26]. Previous data confirmed chemical shifts for HGA [16].

## 4. Conclusions

This study examined how homogentisic acid (HGA) converts into ochronotic pigment, responsible for severe alkaptonuria (AKU) complications. Using various chemical conditions and analysis methods, we characterised pigment formation and its molecular structure. Results show pigment formation is a radical-driven process. Normally, conversion is slow with minimal ring cleavage and produces detectable radicals via EPR spectroscopy, indicating oxidative coupling among rings is key. Testing HGA analogues revealed that the two hydroxyl groups on the aromatic ring are essential; removing one halts the reaction, while side chain modifications have minimal effect, highlighting the diphenol ring’s importance. The pigment appears to be made of heterogeneous particles (11–50 kDa) with a negative charge, suggesting rigid polymers with phenolic ether and biphenyl bonds, consistent with radical coupling. Insights may suggest new strategies to block pigment formation or destabilise it, possibly preventing or reversing ochronosis. However, further studies in tissue-mimicking settings are needed to investigate the role of the tissue environment in pigment deposition.

## Figures and Tables

**Table 1:** SDS-PAGE electrophoresis buffers components for one gel (all products are from Merck KGaA, Darmstadt, Germany).

Stacking Gel	Vol.	Running Gel	Vol.
H_2_O	1.49 mL	H_2_O	1.20 mL
0.5 m Tris-HCl pH 6.8	0.63 mL	1.5 m Tris-HCl pH 8.8	1.25 mL
10% (*m*/*v*) SDS	25 µL	10% (*m*/*v*) SDS	50 µL
Acrylamide/Bis-acrylamide (30%/0.8% *w*/*v*)	0.34 mL	Acrylamide/Bis-acrylamide (30%/0.8% *w*/*v*)	2.50 mL
10% (*m*/*v*) ammonium persulfate	25 µL	10% (*m*/*v*) ammonium persulfate	50 µL
TEMED	2.5 µL	TEMED	5 µL

## Data Availability

The data that support the findings of this study are available from the corresponding author, A.B., upon reasonable request.

## Supplementary Materials

The following supporting information can be downloaded at: https://www.mdpi.com/article/10.3390/ijms26199674/s1.

## Abbreviations

The following abbreviations are used in this manuscript: HGA: homogentisic acid; MW: molecular weight; MWCO: molecular weight cut-off; NMR: nuclear magnetic resonance; EPR: electron paramagnetic resonance: DLS: dynamic light scattering; CP-MAS: Cross-Polarization Magic Angle Spinning.
