# Supplementary material for: Ochronotic Deposition in Alkaptonuria: Semiquinone-Mediated Oxidative Coupling and Metabolic Drivers of Homogentisic Acid Accumulation
Source: Int J Mol Sci. 2025 Oct 3;26(19):9674. doi: 10.3390/ijms26199674 (PMC12524591; doi:10.3390/ijms26199674)
Supplement: Supplementary file 1 [file ijms-26-09674-s001.zip › ijms-3869919-supplementary.pdf]

Supplementary Information

Ochronotic Deposition in Alkaptonuria: Semiquinone-Mediated Oxidative Coupling and Metabolic Drivers of Homogentisic Acid Accumulation

Daniela Grasso [1], Valentina Balloni [1], Camilla Baratto [1], Adele Mucci [2], Annalisa Santucci [1] and Andrea Bernini [1,*]

[1] Department of Biotecnology, Chemistry and Pharmacy, University of Siena, 53100 Siena, Italy; daniela.grasso@student.unisi.it (D.G.); valentina.balloni@unisi.it (V.B.); baratto@unisi.it (M.C.B.); annalisa.santucci@unisi.it (A.S.)
[2] Department of Chemical and Geological Sciences, University of Modena and Reggio Emilia, 41125 Modena, Italy; adele.mucci@unimore.it
* Correspondence: andrea.bernini@unisi.it

NMR monitoring of compounds 11-21 reactions under alkaline catalysis.

Figures S1–S11: NMR spectra of compound 11-21 (5 mM) reaction in alkaline solution.

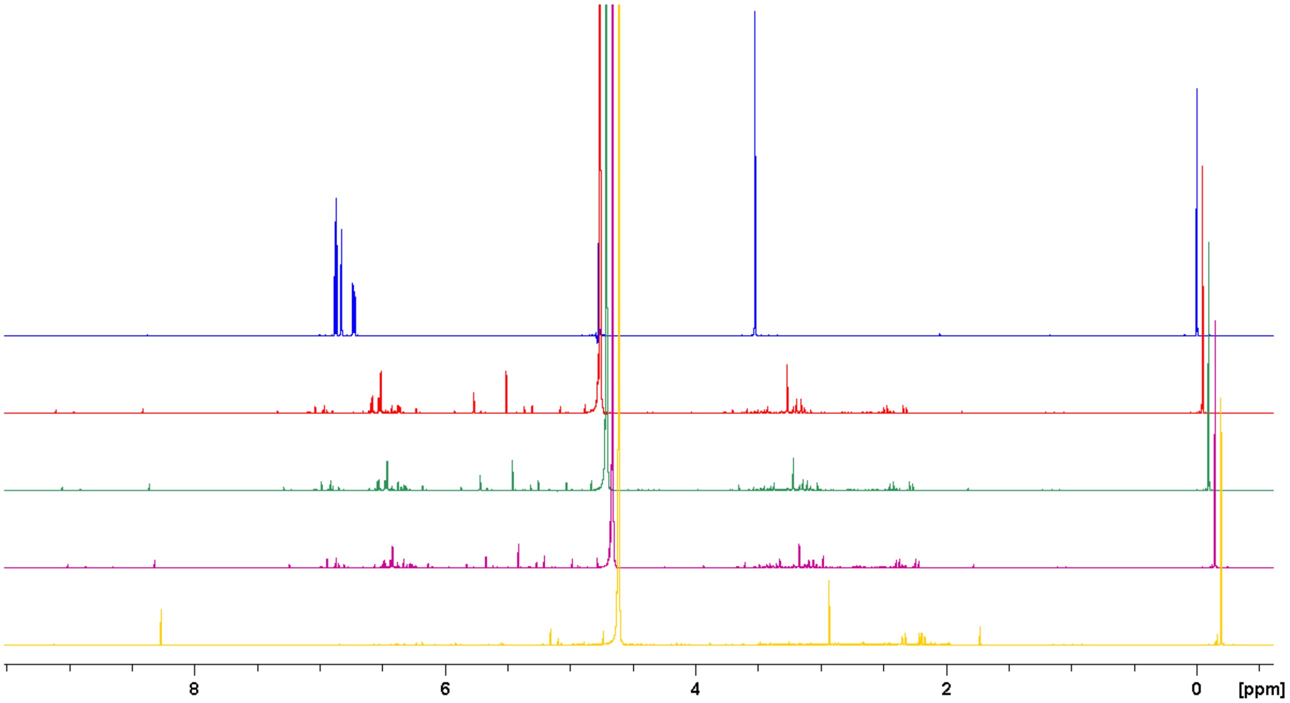

Figure S1. NMR monitoring of **11** (3,4-dihydroxyphenylacetic acid) reaction under alkaline pH at room temperature: from top to bottom: start, 1h, 5 h, 10 h, 1 month. The signals shift significantly due to the increased pH, and the reaction starts immediately, creating byproducts. After 1 month, the starting material is completely consumed.

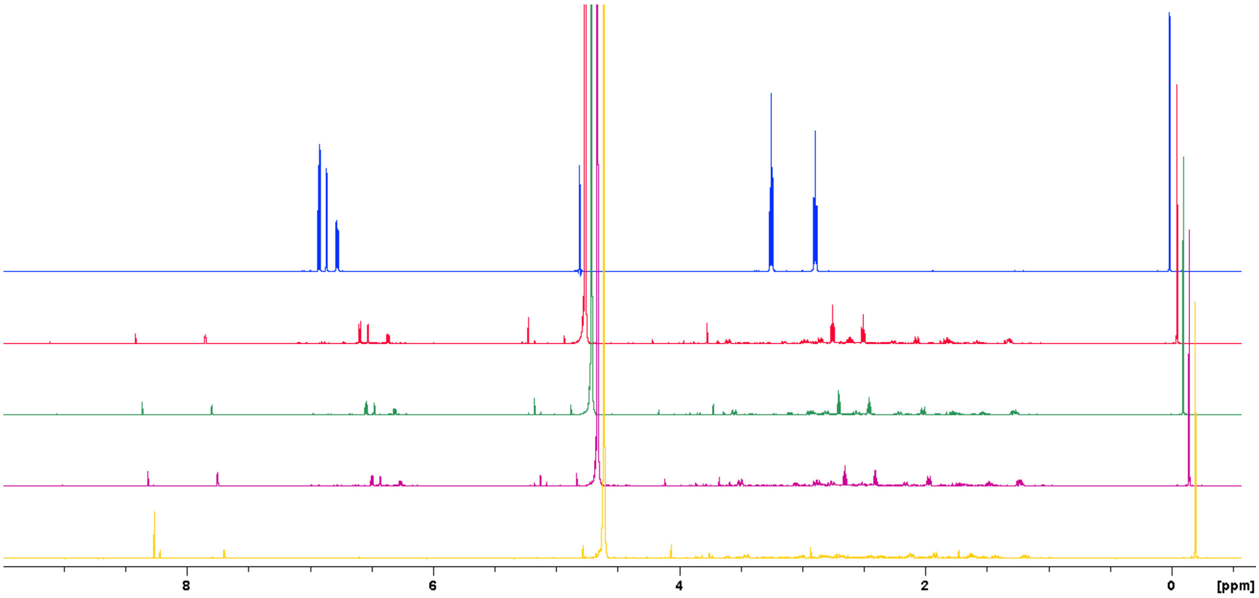

Figure S2. NMR monitoring of **12** (dopamine) reaction under alkaline pH at room temperature: from top to bottom: start, 1h, 5 h, 10 h, 1 month. The signals shift significantly due to the increased pH, and the reaction starts immediately, creating byproducts. After 1 month, the starting material is completely consumed.

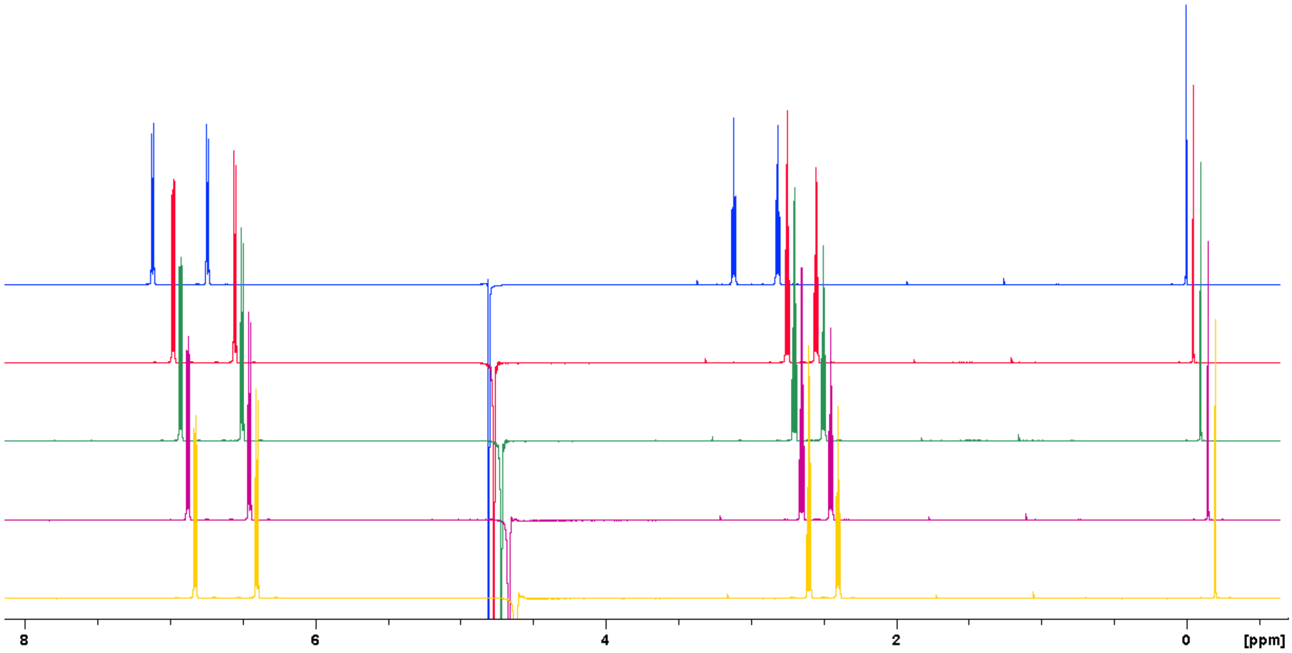

Figure S3. NMR monitoring of **13** (tyramine) reaction under alkaline pH at room temperature: from top to bottom: start, 1h, 5 h, 10 h, 1 month. No reaction was detected, although peaks shifted due to the increased pH.

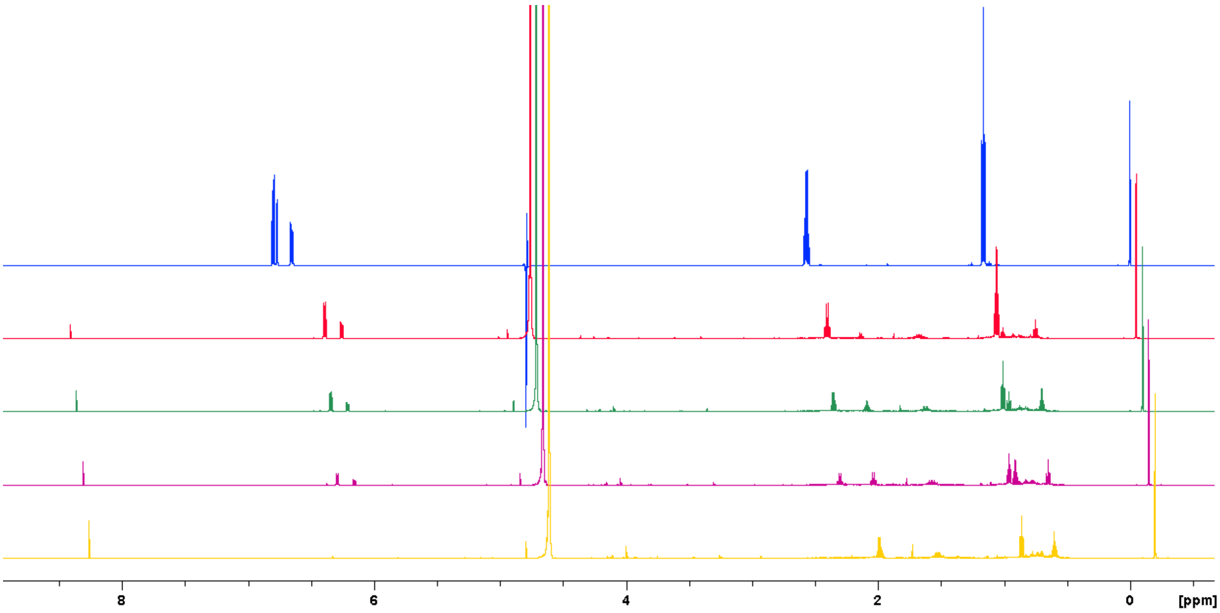

Figure S4. NMR monitoring of **14** (2-ethylbenzene-1,4-diol) reaction under alkaline pH at room temperature: from top to bottom: start, 1h, 5 h, 10 h, 1 month. The signals shift significantly due to the increased pH, and the reaction starts immediately, creating byproducts. After 1 month, the starting material is completely consumed.

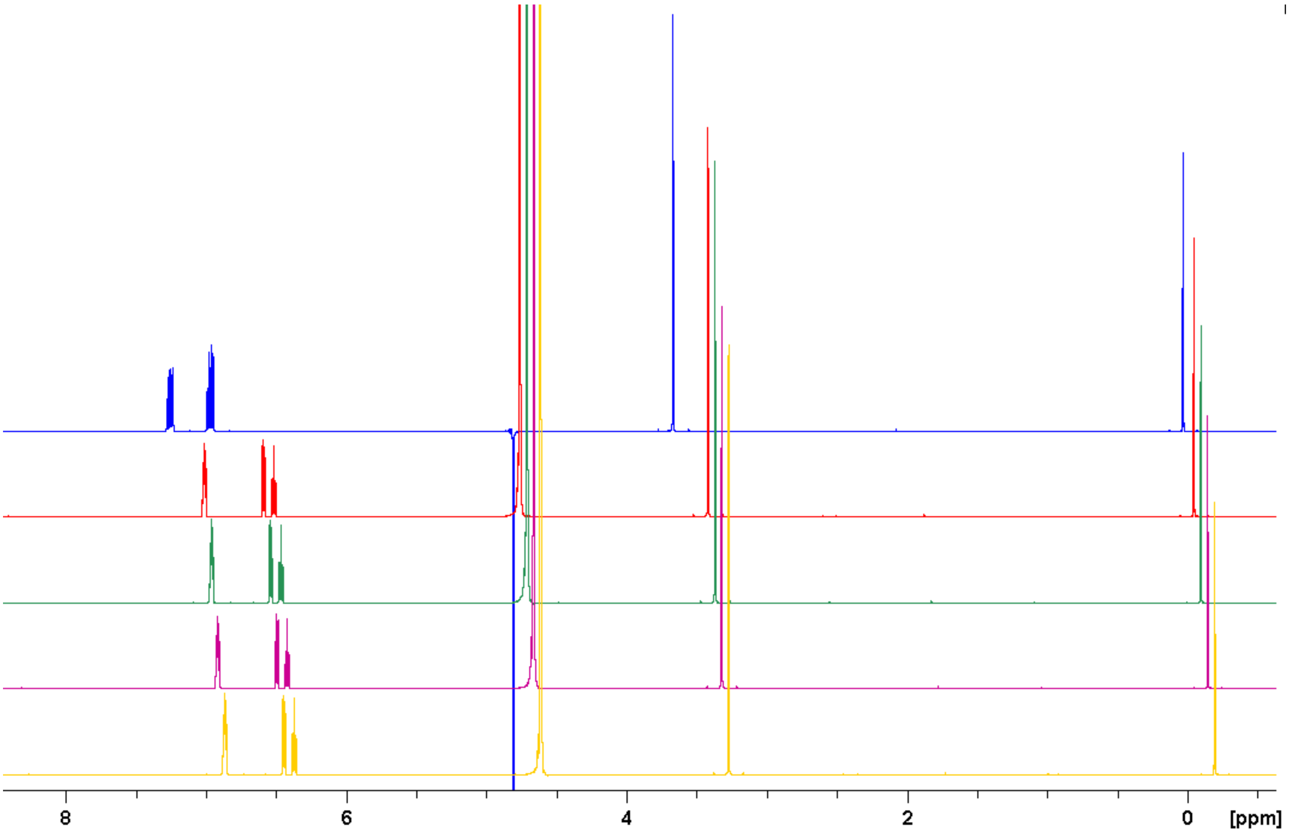

Figure S5. NMR monitoring of **15** (2-hydroxyphenylacetic acid) reaction under alkaline pH at room temperature: from top to bottom: start, 1h, 5 h, 10 h, 1 month. No reaction was detected, although peaks shifted due to the increased pH.

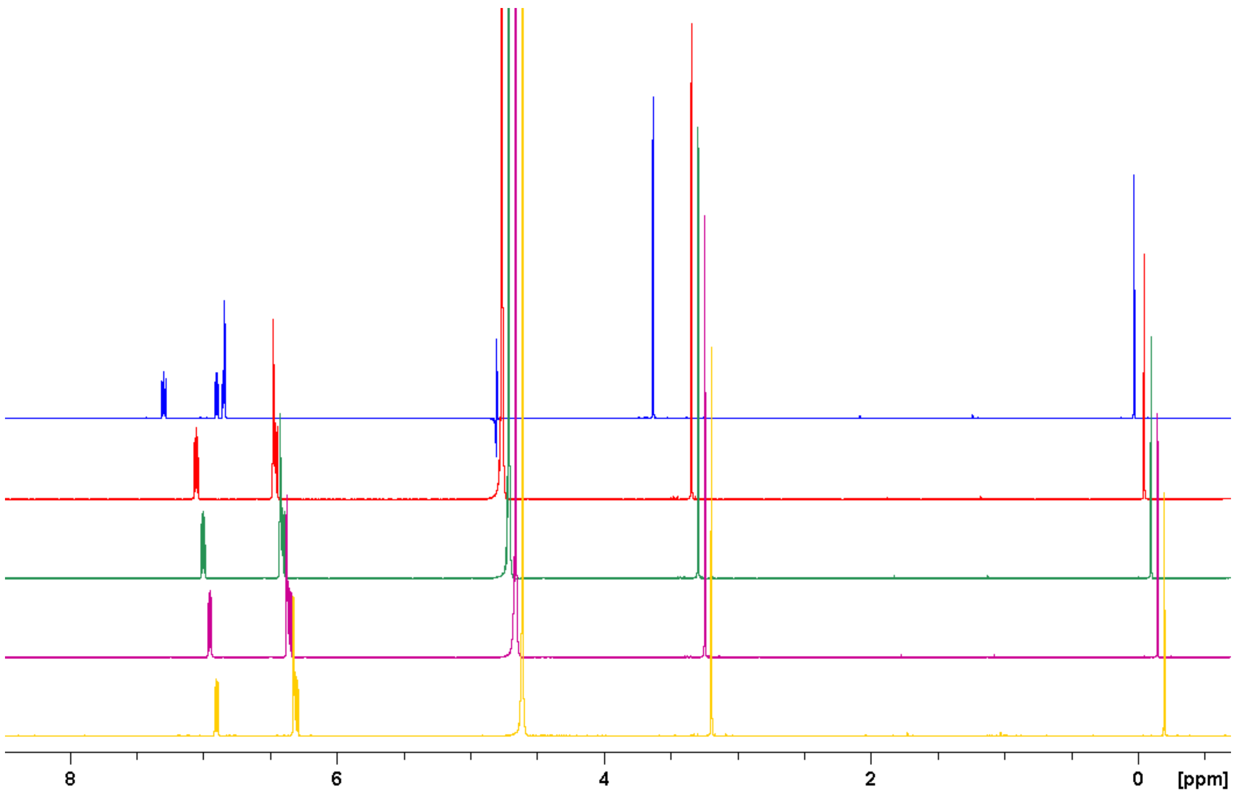

Figure S6. NMR monitoring of **16** (3-hydroxyphenylacetic acid) reaction under alkaline pH at room temperature: from top to bottom: start, 1h, 5 h, 10 h, 1 month. No reaction was detected, although peaks shifted due to the increased pH.

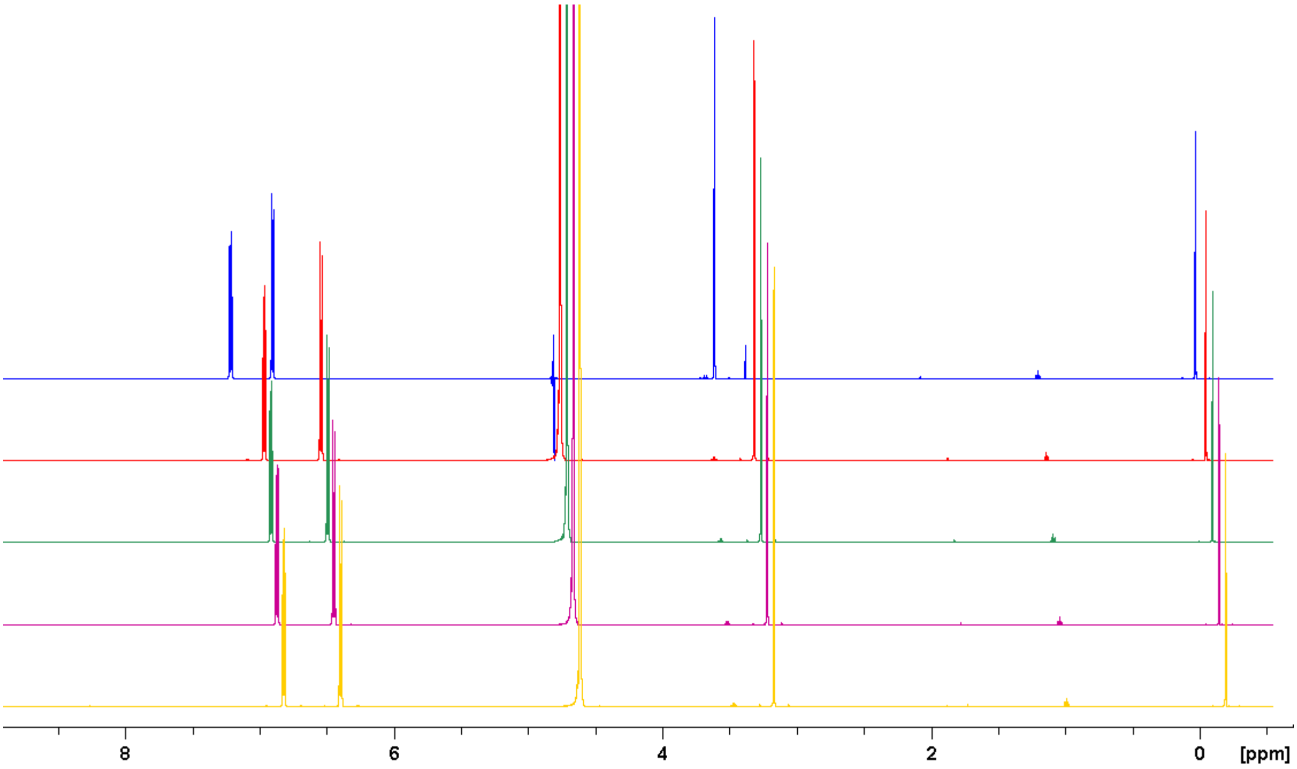

Figure S7. NMR monitoring of **17** (4-hydroxyphenylacetic acid) reaction under alkaline pH at room temperature: from top to bottom: start, 1h, 5 h, 10 h, 1 month. No reaction was detected, although peaks shifted due to the increased pH.

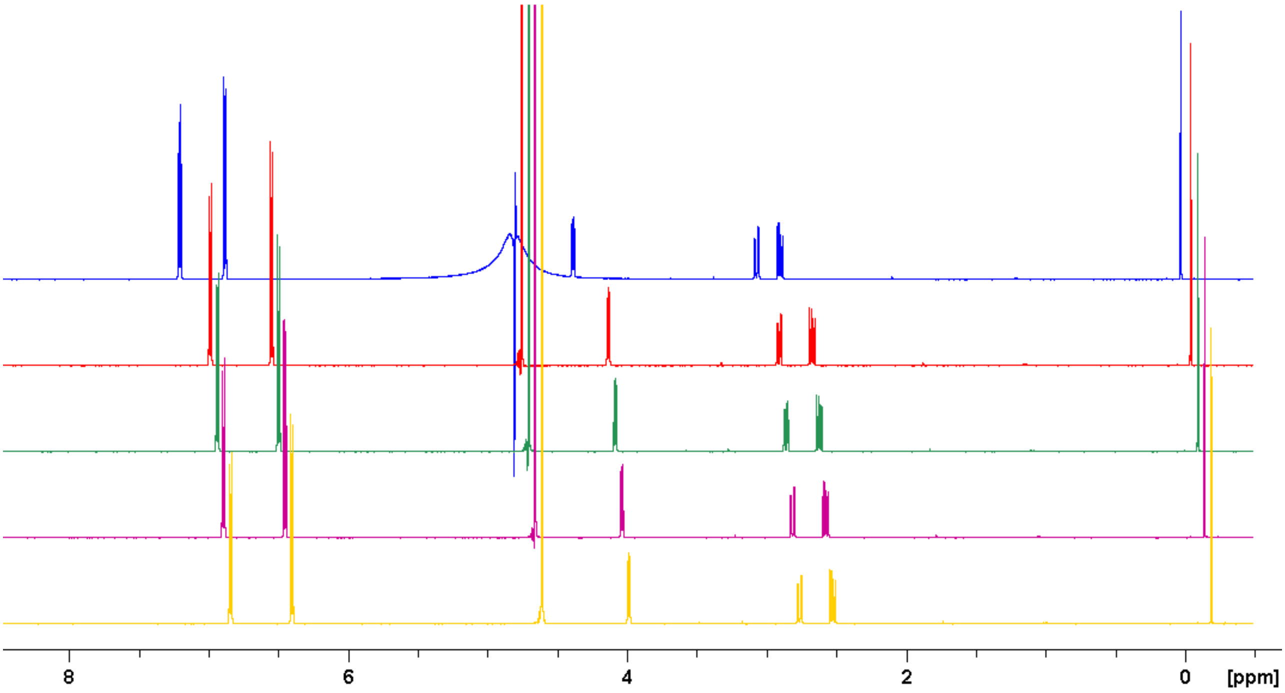

Figure S8. NMR monitoring of **18** (p-hydroxyphenyllactic acid) reaction under alkaline pH at room temperature: from top to bottom: start, 1h, 5 h, 10 h, 1 month. No reaction was detected, although peaks shifted due to the increased pH.

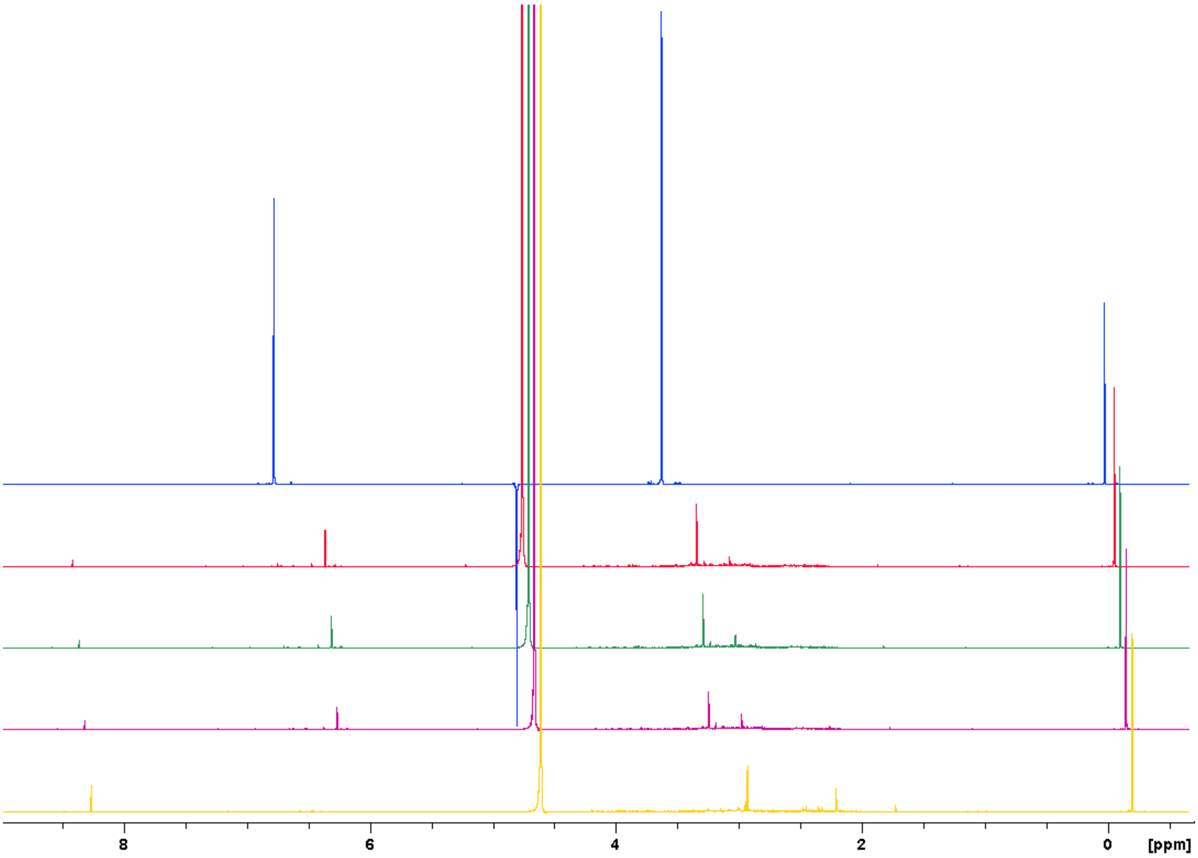

Figure S9. NMR monitoring of **19** (2,5-dihydroxy-1,4-benzendiacetic acid) reaction under alkaline pH at room temperature: from top to bottom: start, 1h, 5 h, 10 h, 1 month. The signals shift significantly due to the increased pH, and the reaction starts immediately, creating byproducts. After 1 month, the starting material is completely consumed.

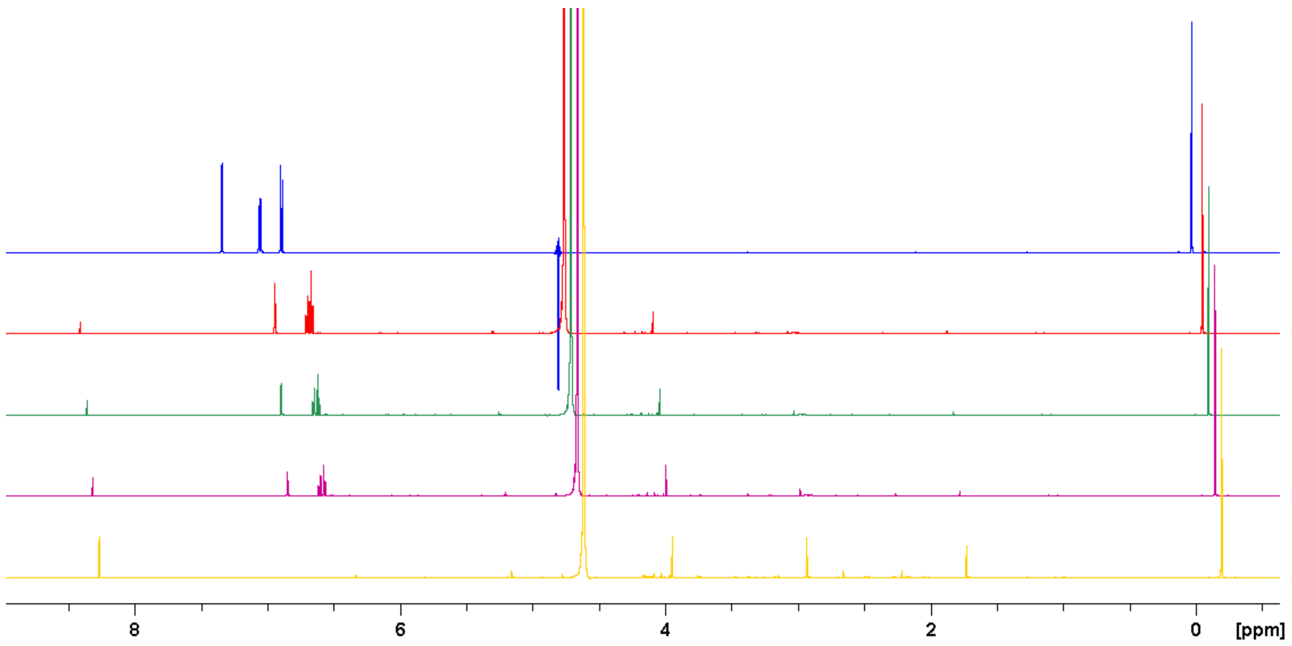

Figure S10. NMR monitoring of **20** (2,5-dihydroxybenzoic acid) reaction under alkaline pH at room temperature: from top to bottom: start, 1h, 5 h, 10 h, 1 month. The signals shift significantly due to the increased pH, and the reaction starts immediately, creating byproducts. After 1 month, the starting material is completely consumed.

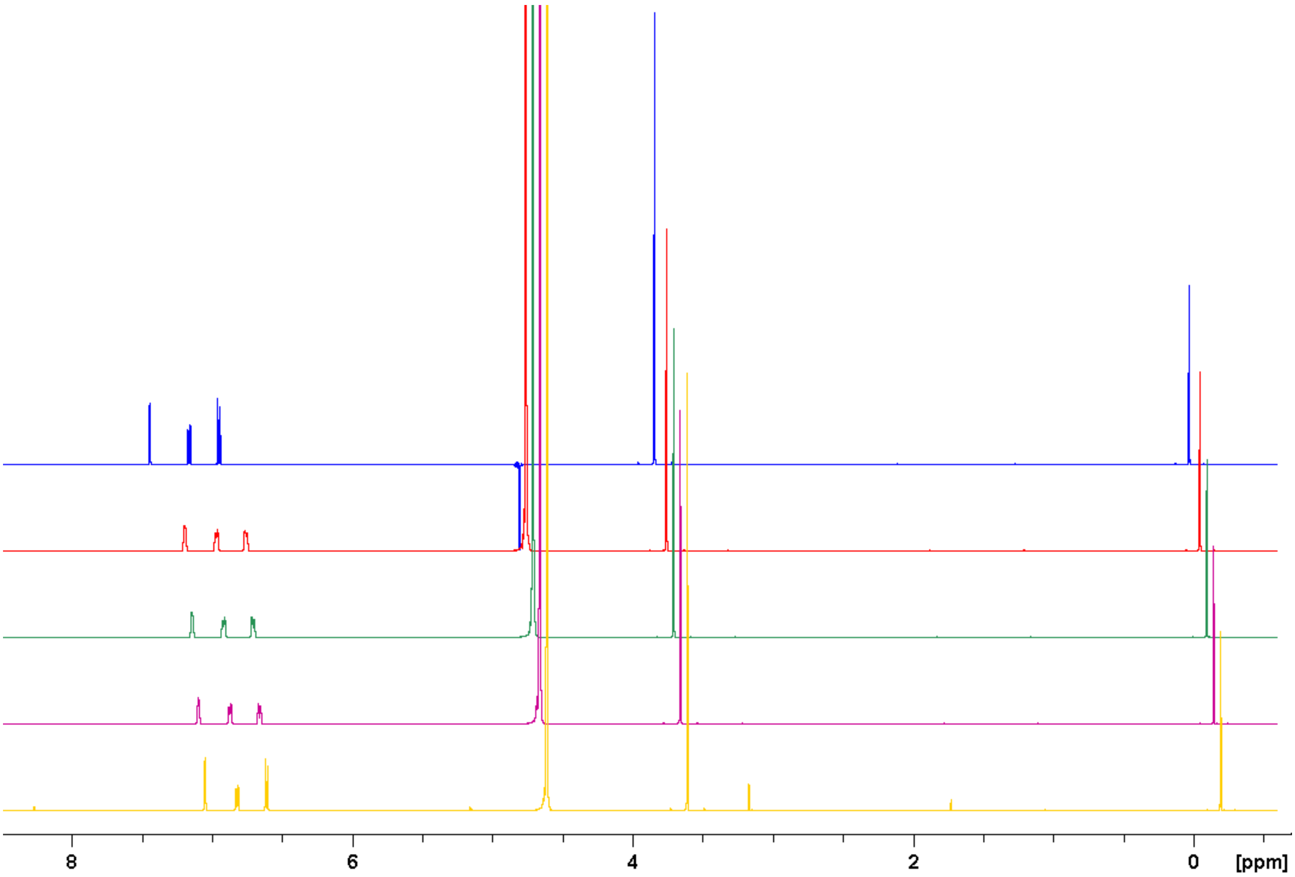

Figure S11. NMR monitoring of 21 (2-hydroxy-5-methoxybenzoic acid) reaction under alkaline pH at room temperature: from top to bottom: start, 1h, 5 h, 10 h, 1 month. No reaction was detected, although peaks shifted due to the increased pH.

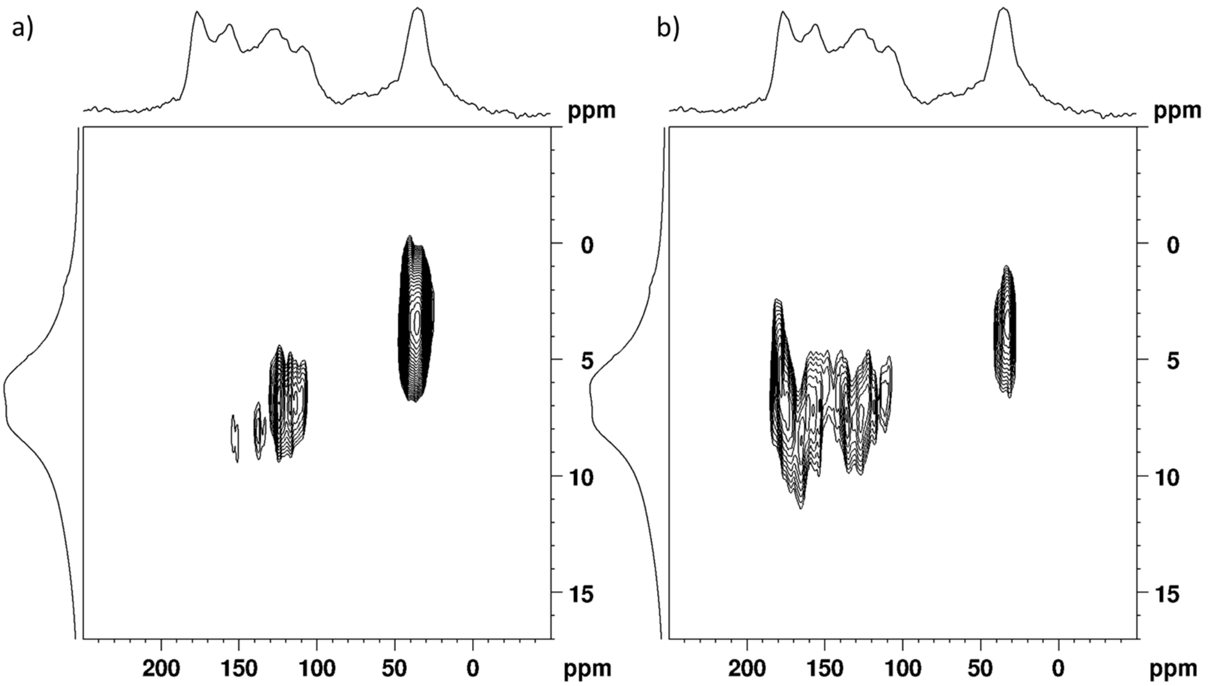

Figure S12. Hetero-correlated C,H FSLG 2D NMR experiments. A 0.2 ms contact time (a) permits to detect directly bound C-H pairs, while 1 ms contact time (b) permits to detect also quaternary carbons.
